# Production of Bioactive Porcine Lactoferrin through a Novel Glucose-Inducible Expression System in *Pichia pastoris*: Unveiling Antimicrobial and Anticancer Functionalities

**DOI:** 10.3390/ijms25031818

**Published:** 2024-02-02

**Authors:** Chih-Ching Yen, Pei-Ying Wu, Huan Ou-Yang, Hsiao-Ling Chen, Kowit-Yu Chong, Ro-Lin Chang, Chuan-Mu Chen

**Affiliations:** 1 Department of Internal Medicine, China Medical University Hospital, College of Health Care, China Medical University, Taichung 404, Taiwan; d5210@mail.cmuh.org.tw; 2 Department of Life Sciences, Ph.D. Program in Translational Medicine, National Chung Hsing University, Taichung 402, Taiwan; aa0929051229@gmail.com (P.-Y.W.); huan4096052151@alumni.nchu.edu.tw (H.O.-Y.); 3 Department of Biomedical Science, Da-Yeh University, Changhua 515, Taiwan; hlchenbell@gmail.com; 4 Department of Medical Biotechnology and Laboratory Science, College of Medicine, Chang Gung University, Taoyuan 333, Taiwan; kchong@mail.cgu.edu.tw; 5 Hyperbaric Oxygen Medical Research Laboratory, Bone and Joint Research Center, Chang Gung Memorial Hospital, Taoyuan 333, Taiwan; 6 The iEGG and Animal Biotechnology Center, Rong Hsing Research Center for Translational Medicine, National Chung Hsing University, Taichung 402, Taiwan

**Keywords:** lactoferrin, *G1* promoter, *Pichia pastoris*, fermentation, tangential-flow ultrafiltration, iron-binding, antimicrobial, anticancer

## Abstract

Lactoferrin (LF) stands as one of the extensively investigated iron-binding glycoproteins within milk, exhibiting diverse biological functionalities. The global demand for LF has experienced consistent growth. Biotechnological strategies aimed at enhancing LF productivity through microbial expression systems offer substantial cost-effective advantages and exhibit fewer constraints compared to traditional animal bioreactor technologies. This study devised a novel recombinant plasmid, wherein the *AOX1* promoter was replaced with a glucose-inducible *G1* promoter (P_G1_) to govern the expression of recombinant porcine LF (rpLF) in *Pichia pastoris* GS115. High-copy-number P_G1_-rpLF yeast clones were meticulously selected, and subsequent induction with 0.05 g/L glucose demonstrated robust secretion of rpLF. Scaling up production transpired in a 5 L fermenter, yielding an estimated rpLF productivity of approximately 2.8 g/L by the conclusion of glycerol-fed fermentation. A three-step purification process involving tangential-flow ultrafiltration yielded approximately 6.55 g of rpLF crude (approximately 85% purity). Notably, exceptional purity of rpLF was achieved through sequential heparin and size-exclusion column purification. Comparatively, the present glucose-inducible system outperformed our previous methanol-induced system, which yielded a level of 87 mg/L of extracellular rpLF secretion. Furthermore, yeast-produced rpLF demonstrated affinity for ferric ions (Fe^3+^) and exhibited growth inhibition against various pathogenic microbes (*E. coli*, *S. aureus*, and *C. albicans*) and human cancer cells (A549, MDA-MB-231, and Hep3B), similar to commercial bovine LF (bLF). Intriguingly, the hydrolysate of rpLF (rpLFH) manifested heightened antimicrobial and anticancer effects compared to its intact form. In conclusion, this study presents an efficient glucose-inducible yeast expression system for large-scale production and purification of active rpLF protein with the potential for veterinary or medical applications.

## 1. Introduction

Lactoferrin (LF), a glycoprotein binding iron with a molecular weight ranging from 78 to 80 kDa, belongs to the transferrin superfamily [1]. LFs identified in various species share a commonality of 689 to 702 amino acid residues, exhibiting substantial sequence homology. For instance, there is a 69.3% identity and 83.4% similarity between human LF (hLF) and bovine LF (bLF) as well as a 70.3% identity and 82.6% similarity between hLF and porcine LF (pLF). Primarily secreted during an animal’s lactation period, notably in colostrum, LF secretion reaches approximately 7 g/L. Additionally, mucosal fluids such as saliva, tears, bile, pancreatic juice, gastric juice, bronchial, and uterine secretions contribute to LF presence [2]. In inflammatory conditions, LF is released from the secondary granules of neutrophils. Beyond its role in iron transport, LF encompasses a broad spectrum of biological functions, including anti-inflammatory, anticancer, antimicrobial, antioxidant, and immunomodulatory effects [3,4,5,6]. The diverse functionalities of LF position it as a prospective antibiotic, anticancer therapeutic, and a potential addition to food or feed [7,8,9]. Concurrently, the investigation of LF-derived peptides has emerged as a distinct area of LF research. Renowned for their antimicrobial properties [10], LF-derived peptides exhibit diverse microbicidal activities against enteroaggregative *Escherichia coli* [11], *Giardia intestinalis* [12], and Crohn’s disease [13], showcasing promise for non-antibiotic therapeutic interventions.

The expression of hLF in *Aspergillus oryzae* marked the inaugural instance of artificially producing recombinant lactoferrin (rLF) [14]. Subsequently, diverse recombinant lactoferrins have been generated at varying yields across a spectrum of biological systems, including bacteria [15], yeast [16], insect larvae [17], and plants [18]. Yeast, particularly *Saccharomyces cerevisiae* and *Pichia pastoris*, stands out as the predominant host for lactoferrin expression, leveraging its rapid growth, ease of manipulation, and high expression capabilities. Notably, yeast has been employed to produce hLF, reaching levels of 1.5~2.0 mg/L through the chelatin promoter in *S. cerevisiae* AB116 [19] and Tibetan sheep lactoferrin exceeding 60 mg/L using the *alcohol oxidase I* (*AOX1*) promoter in *P. pastoris* GS115 [20]. The choice of vector promoter significantly influences the quantity of lactoferrin expression, with two categories of promoters commonly employed in *P. pastoris*: non-methylotrophic promoters (e.g., glyceraldehyde-3-phosphate dehydrogenase gene—*GAP* [21], translation elongation factor 1-α gene—*TEF* [22], and glucose-limit-inducible genes—*G1* to *G8* [23]) and methylotrophic promoters (e.g., *AOX1*, *AOX2*, and glutathione-dependent formaldehyde dehydrogenase gene—*FLD1*) [24,25,26,27]. While the non-methylotrophic *GAP* promoter allows for constitutive protein production, it poses challenges such as unregulated expression, leaky protein expression, and low secretion efficiency for proteins exceeding 30 kD [22], rendering it unsuitable for high-density cultivation. The *G1* promoter, surpassing the *GAP* promoter by over twofold in glucose-limited fed-batch cultures without methanol, emerges as a more favorable alternative [23].

In this study, we engineered a novel pPICZαC-P_G1_-pLF plasmid, replacing the original *AOX1* promoter in our prior plasmid (pPICZαC-AOX1-rpLF [28]) with a *G1* promoter sequence. The resultant recombinant porcine lactoferrin protein was produced using a bench-top fermenter, and its purification was assessed. Subsequently, we conducted iron-binding, antimicrobial, and anticancer assays to delineate the biological functions of rpLF protein. Additionally, we subjected rpLF protein to hydrolysis by gastrointestinal enzymes and compared the functional distinctions among bLF, pLF, and the principal rpLF hydrolysate (rpLFH).

## 2. Results

Previously, we developed the pPICZαC-rPLF plasmid for methanol-inducible rpLF expression in yeast under the *AOX1* promoter [28]. In this study, we modified this construct by replacing the *AOX1* promoter (cut by *Bgl*II and *Hind*III) with the *G1* promoter (cut by *Bam*H1 and *Hind*III), resulting in a novel 5.8 kb plasmid, pPICZαC-P_G1_-rpLF (Figure S1). After confirming the correct reading frame for rpLF expression through sequencing, we introduced the plasmid into *Pichia pastoris* for characterizing protein expression, purification, and biological functions.

### 2.1. Small- and Large-Scale Expression of rpLF in P. pastoris GS115

**Figure 1:**
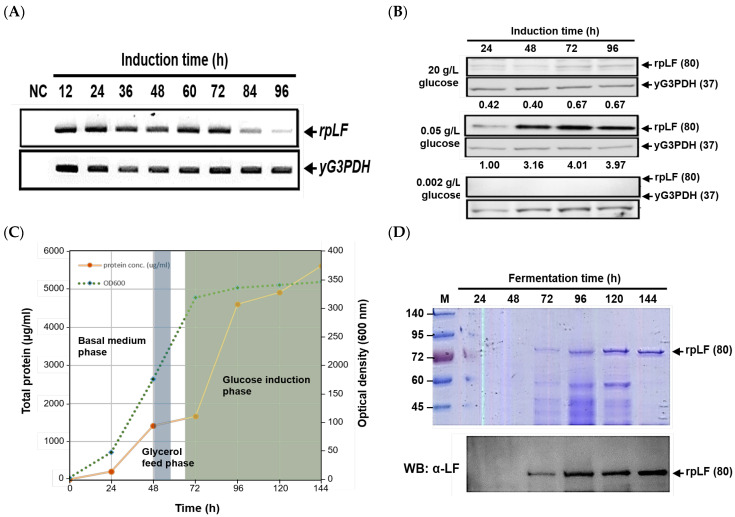
Characterization of rpLF expression in shaker flasks and bench-top fermenters. (**A**) RT-PCR analysis of time-course rpLF mRNA change in shaker flasks. (**B**) Analysis of rpLF protein secretion. The yeast cultures were induced with 20, 0.05, and 0.001 g/L glucose for rpLF production, respectively. Culture supernatants were collected daily for Western blot analysis of rpLF secretion. In (**A**,**B**), yG3PDH served as a loading control. Relative rpLF protein secretions, normalized to yG3PDH, are indicated below the images and compared with rpLF secretion at 24 h of 0.05 g/L glucose induction. (**C**) Monitoring of total protein secretion in the culture supernatant and the yeast growth profile in a fed-batch yeast culture. The culture was conducted in a bench-top fermenter (5-L scale) for 144 h, with glycerol and glucose fed at 48–56 h and 70–144 h, respectively. (**D**) SDS-PAGE and Western blot identification of rpLF protein expression throughout the fermentation process.

Following transformation, positive transformants with high-copy-number integration into the yeast chromosome were identified using slot-blot DNA hybridization. Expression and secretion of rpLF into the culture medium were confirmed upon glucose induction (Figure S2). A selected positive transformant (clone #13 in Figure S2) was further investigated to characterize the transcriptional profile of the *rpLF* gene and assess the impact of glucose concentrations on rpLF expression. As depicted in Figure 1A, *rpLF*-specific transcription became detectable at 12 h post glucose induction, persisting until 72 h before gradually diminishing. At the protein level (Figure 1B), induction with 0.05 g/L glucose resulted in the highest rpLF secretion, the next highest was by 20 g/L glucose induction, and almost undetectable levels occurred with 0.001 g/L glucose. Moreover, significant rpLF secretion commenced 48 h after 0.05 g/L glucose addition, peaked at 72 h, and continued until 96 h.

The scaled-up production of rpLF was conducted in a 5 L benchtop fermenter with glycerol-fed batch fermentation (Figure 1C). Cell densities exhibited rapid growth within the initial 48 h, followed by exponential growth after glycerol feeding, reaching a stationary phase shortly after glucose induction. At this point, OD_600_ approached approximately 350. Extracellular protein concentrations increased concomitantly with growing cell densities, experiencing a sharp rise after one day of glucose feeding and a gradual increase until the end of fermentation. The estimated extracellular protein concentration at the endpoint was approximately 5.6 g/L. SDS-PAGE examination illustrated the secretion pattern, confirming rpLF as a major protein in the extracellular medium with an approximate molecular weight of 80 kD (Figure 1D). Notably, the initial purity of rpLF exceeded 50% at the conclusion of fermentation. Hence, the estimated productivity of rpLF throughout this glycerol-fed fermentation process was at least 2.8 g/L before purification. 

### 2.2. Purification of rpLF

In this study, a 4.5 L yeast culture was harvested, and approximately 2.9 L of culture supernatant underwent rpLF purification after yeast pellet removal. The entire purification, completed in one day, involved three ultrafiltration steps. As summarized in Table 1, the initial culture supernatant, filtered using a 0.45 μm hollow fiber cartridge, yielded a 2.4 L filtrate with rpLF at 4.3 g/L and approximately 52% purity. The filtrate volume was further reduced to about 1.4 L with a 100 kD cassette, increasing rpLF concentration to 7.3 g/L and purity to nearly 68%. The second step resulted in a 5% loss of rpLF protein. In the third step, a 30 kD cassette condensed the 100 kD filtrate to 240 mL, further enhancing rpLF concentration to approximately 23 g/L and purity to 85%. However, nearly half of the rpLF protein was lost during this step.

**Figure 2:**
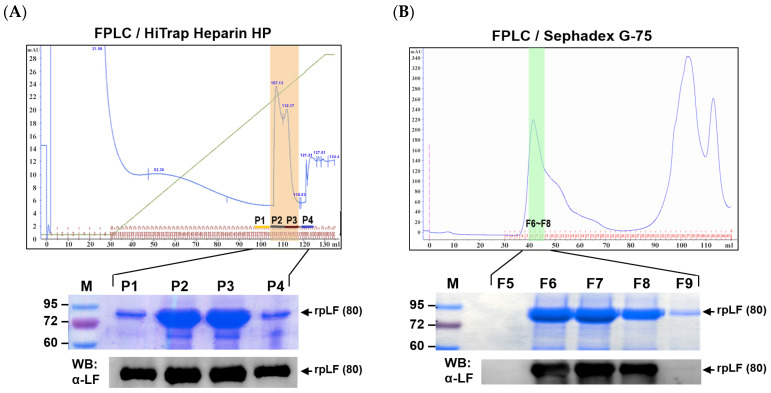
Efficient purification of rpLF protein. Following sequential ultrafiltration steps outlined in Table 1, the rpLF crude underwent additional purification through heparin affinity (**A**) and Sephadex G-75 column (**B**) techniques. Collected fractions were analyzed using SDS-PAGE and Western blot to assess rpLF protein purity.

Aliquots of the rpLF crude underwent column purification for the subsequent functional assays. Utilizing a 5 mL heparin affinity column (Figure 2A), rpLF purity increased slightly (approximately 91%), with a major band of rpLF and a minor band near 70 kD visible in the gel. Pooled rpLF fractions (P1 to P4 in Figure 2A) were further separated with a Sephadex G-75 size-exclusion column (Figure 2B). Fractions corresponding to the major protein peak, examined by SDS-PAGE, revealed a distinct rpLF band with approximately 94% purity (F6 to F8 in Figure 2B).

### 2.3. Iron (Fe^3+^)-Binding Activity of rpLF

**Figure 3:**
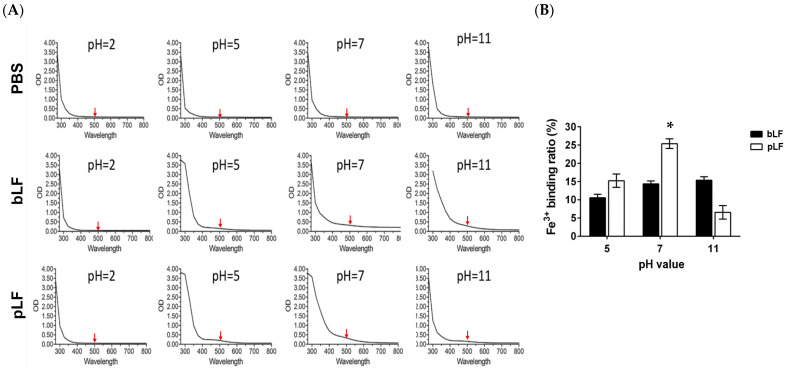
Iron-binding assay. (**A**) Spectra of the iron-binding assays conducted at different pH values, utilizing a protein concentration of 5 mg/mL and optical density (OD) measurement across wavelengths from 280 nm to 680 nm. Iron-binding ratios were determined by comparing OD500 units of protein-bound iron concentrations to initial iron concentrations. (**B**) Comparative analysis of the iron-binding capabilities of bLF and rpLF at pH 5, pH 7, and pH 11. * *p* < 0.05.

Next, rpLF was characterized for its iron-binding activity and compared to commercial bLF. As shown in Figure 3, rpLF displayed higher iron-binding capacities than commercial bLF at pH 5 and pH 7 (*p* < 0.05) but had a lower iron-binding performance at pH 11. However, neither rpLF nor commercial bLF showed notable iron-binding reactions at pH 2.

### 2.4. Simulated Gastrointestinal Digestion of rpLF

**Figure 4:**
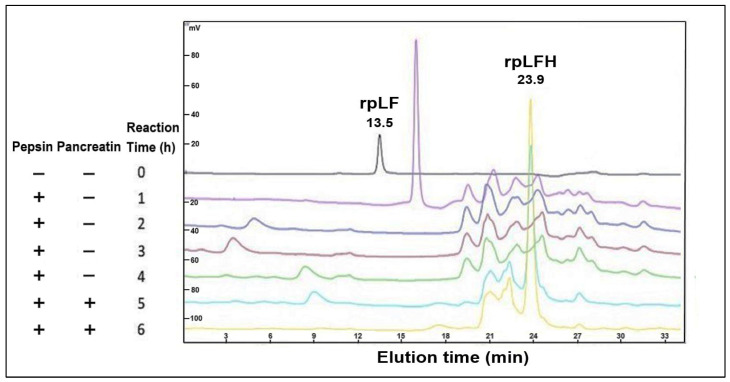
HPLC analysis was performed on rpLF following simulated gastrointestinal digestion. The analysis was carried out at a flow rate of 0.5 mL/min, with peptides below 3 kD expected to elute after 20 min. Elution times for both rpLF and rpLFH were noted, and the rpLFH peak was collected for subsequent antimicrobial and anticancer assays.

The current investigation extended its examination to simulate gastrointestinal digestion of rpLF using pepsin and pancreatin. HPLC analysis demonstrated that the intact rpLF protein (eluted at 13.5 min) underwent extensive hydrolysis into multiple short peptides within 6 h, evident by elution times exceeding 20 min (Figure 4). Notably, a prominent peak in the rpLF hydrolysate (rpLFH, eluted at 23.9 min) was isolated for subsequent functional comparisons with the intact rpLF protein.

### 2.5. Antimicrobial Activities of rpLF and rpLFH

**Figure 5:**
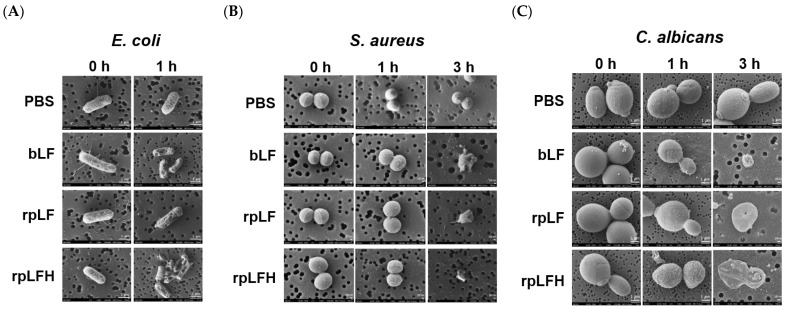
Scanning electron microscopic images showing the antimicrobial effects of bLF, rpLF, and rpLFH on (**A**) *E. coli*, (**B**) *S. aureus*, and (**C**) *C. albicans*. In this study, 5 mg/mL of bLF and rpLF as well as 500 μg/mL of rpLFH were used in antimicrobial assays. The images were captured with 30,000-fold magnification. Scale bars represent 1 μm.

The antimicrobial efficacy of rpLF was assessed against *Escherichia coli* ATCC 25922 (*E. coli*; Gram-negative bacterium), *Staphylococcus aureus* ATCC 25923 (*S. aureus*; Gram-positive opportunistic pathogen), and *Candida albicans* ATCC 14053 (*C. albicans*; fungal pathogen). At a concentration of 5 mg/mL, rpLF exhibited rapid damage to *E. coli* within 1 h (Figure 5A) and significant antimicrobial activity against both *S. aureus* (Figure 5B) and *C. albicans* (Figure 5C) within 3 h. Notably, rpLF demonstrated comparable antimicrobial effects to commercial bLF at the same concentration. Remarkably, rpLFH, at a ten-fold lower concentration (500 μg/mL), induced similar damage to these microbes, demonstrating efficacy on par with rpLF and commercial bLF at 5 mg/mL. Microscopic analysis revealed visible damage to the plasma membranes of the microbes, leading to substantial shrinkage (Figure 5).

Consistent with microscopic observations (Figure 5), rpLF exhibited a minimum inhibitory concentration (MIC) of 720 μg/mL against *E. coli* and *C. albicans* and an MIC of 960 μg/mL against *S. aureus*. Additionally, rpLF displayed a minimum bactericidal concentration (MBC) exceeding 960 μg/mL for all tested microorganisms. Commercial bLF showed lower MICs compared to rpLF but had MBCs > 960 μg/mL for the tested microbes. Furthermore, rpLFH exhibited significantly lower MIC and MBC values than intact rpLF. The MIC and MBC of rpLFH were 120 and 240 μg/mL for *E. coli*, 180 and 360 μg/mL for *S. aureus*, and 240 and 480 μg/mL for *C. albicans*, respectively (Table 2).

### 2.6. Anticancer Activities of rpLF and rpLFH

**Figure 6:**
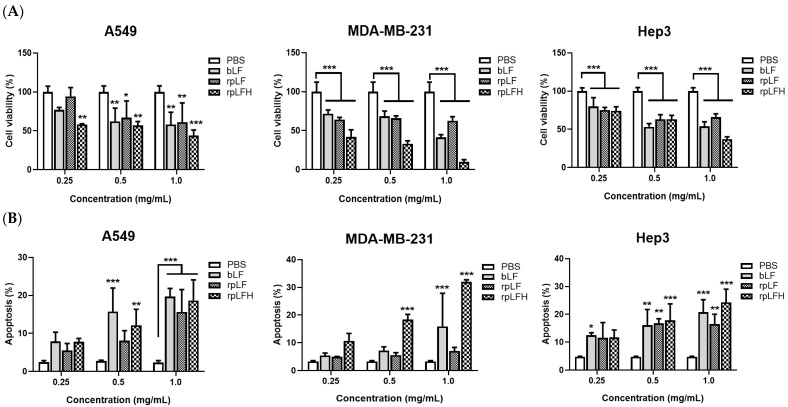
Anticancer effects of bLF, rpLF, and rpLFH on A549, MDA-MB-231, and Hep3B cells. (**A**) Cell viability assay. (**B**) Apoptotic assay. In this study, cells were treated with different concentrations of bLF, rpLF, and rpLFH for 48 h in triplicate for three independent experiments. Statistical analysis was conducted using two-way ANOVA and Dunnett’s multiple comparisons test. Statistical symbols: * *p* < 0.05, ** *p* < 0.01, and *** *p* < 0.001 compared with the PBS control.

The anticancer effects of rpLF and rpLFH were evaluated on A549, MDA-MB-231, and Hep3B cells using cell viability and apoptosis assays. As depicted in Figure 6, both rpLF and rpLFH along with commercial bLF exhibited substantial inhibition of cell viability (Figure 6A) and induced significant apoptosis (Figure 6B) in these tumor cell lines in vitro. Notably, there were no significant differences observed between rpLF and commercial bLF in terms of their impact on cell viability or apoptosis induction in tumor cells. However, rpLFH demonstrated increased cytotoxicity compared to intact rpLF and bLF in A549, MDA-MB-231, and Hep3B cells, especially at a concentration of 1 mg/mL, where rpLFH caused a more than 50% reduction in cell viability for these cancer cells.

## 3. Discussion

The utilization of yeast expression systems for large-scale recombinant protein production has been extensively explored [29], revealing a positive correlation between expressed protein levels and integrated target gene copy numbers in the host chromosome [30]. Common yeast hosts include *S. cerevisiae* and the methylotrophic *P. pastoris*, with *P. pastoris* generally exhibiting higher secretion efficiency, making it more suitable for industrial-scale recombinant protein production than *S. cerevisiae*. Apart from host factors, the choice of transcriptional promoters upstream of target genes significantly influences final protein productivity in yeast.

While the *AOX1* promoter is frequently employed for target gene transcription in *P. pastoris*, its reliance on methanol for inducing protein translation limits its applicability for industrial protein production due to methanol’s toxic and highly flammable nature. An alternative non-methylotrophic and glucose-limit-inducible *G1* promoter was thoroughly investigated by Prielhofer et al. [23]. They identified six novel promoter candidates (*G1*, *G3*, *G4*, *G6*, *G7*, and *G8*) and demonstrated that the *G1* promoter drove a 2.4-fold higher secretion of human serum albumin (HSA) than the widely-used *GAP* promoter at the end of glycerol-fed batch fermentation [23].

In this study, we replaced the original *AOX1* promoter region of a previously constructed recombinant plasmid (pPICZαC-rPLF) [28] with the *G1* promoter, creating a new construct, pPICZαC-P_G1_-rpLF (Figure S1). Our findings indicate that rpLF was secreted at a high level, approximately 2.8 g/L, at the end of fed-batch fermentation, significantly surpassing the previous 87 mg/L achieved with methanol induction in shaker flasks [28]. Notably, LF from various species, including goats, sheep, yaks, monkeys, pigs, horses, and humans, has been successfully expressed in *P. pastoris* [16,20,30,31,32,33,34,35]. While the *AOX1* promoter was predominantly used, LF expression levels ranged from a few milligrams to several tens of milligrams per liter of yeast broth in small-scale shaker flask cultivations. Through batch fermentation, Iglesias-Figueroa et al. [36] reported that the expression yield of bLF under the AOX1 promoter reached 3.5 g/L, demonstrating a significant increase in LF productivity compared to shaker flask cultivations.

The *G1* promoter-driven protein expression is intricately governed by the residual glucose concentration in the culture media. Prielhofer et al. [23] demonstrated that the *G1* promoter exhibits full activity at glucose concentrations below 0.05 g/L, with repression observed at 20 g/L glucose. Similar outcomes were observed in our study, where the optimal rpLF secretion occurred with 0.05 g/L glucose induction in small-scale shaker flask cultivations (Figure 1B). Maximum rpLF secretion was evident 48 h after glucose addition, indicating that residual glucose at this point was less than 0.5 g/L, and most glucose was utilized for inducing rpLF translation. In contrast, effective rpLF induction was unattainable at 20 g/L glucose, possibly due to extensive glucose consumption in yeast growth. Moreover, at 0.001 g/L concentration, glucose might be rapidly depleted after addition, leaving no residual glucose for rpLF translation induction.

Similarly, in large-scale fermenter cultivation (Figure 1C), rpLF secretion was undetectable during the initial basal medium phase (0–48 h), underscoring the stringent regulation of the *G1* promoter. With glycerol feeding, exponential yeast growth persisted, entering a stationary phase shortly after the initiation of the glucose induction phase (70–144 h). Throughout the glucose induction phase, yeast growth remained at maximum density, and rpLF secretion gradually increased until the completion of fermentation, underscoring the high efficiency of the entire fermentation process for rpLF production.

Protein secretion into the media simplifies the purification process for yeast expression systems. In this study, rpLF purification was carried out using tangential-flow ultrafiltration. The culture supernatant volume was reduced over ten times, resulting in a final protein solution containing 23.1 mg/mL of rpLF with a purity of nearly 85%, and over half of the rpLF protein was recovered (Table 1). These procedures enhance purification efficiency, and the entire process can be completed in a single day. Choi et al. [31] employed a similar strategy for hLF purification, reporting a recovery of 21% after a series of filtration steps.

Ammonium sulfate precipitation (ASP) has historically served as the initial LF purification step, but most studies lack detailed characterization of this phase [30,33,36]. Li et al. [20] reported a 91.1% activity yield using ASP for sheep LF (sLF) purification, although the data were obtained from a small yeast culture volume (6 mL). While ASP allows bulk protein precipitation, drawbacks include the need for large quantities of AS, increased volume post-AS addition, and co-precipitation of unwanted proteins with the target protein, limiting its suitability for industrial purification.

Heparin-affinity column (HAC) chromatography was employed in our experiments to further purify rpLF from 85% to 91% purity (Figure 2A). Although effective for LF purification, HAC utilizes heparin, an animal-derived component with contamination risks, making it less suitable for industrial purification. Following HAC, size-exclusion column (SEC) chromatography raised rpLF purity to 94% (Figure 2B). SEC or gel filtration is commonly employed as the final step in protein purification. Additionally, a polyhistidine tag was incorporated at the C-terminus of the rpLF reading frame; however, purification using a Ni^2+^-NTA affinity column proved ineffective, suggesting that the histidine tag may not be exposed to immobilized Ni^2+^.

Most studies utilizing *P. pastoris* for the production of various recombinant LFs have consistently confirmed their analogous iron-binding properties to natural LFs, underscoring the structural accuracy and stability of recombinant LFs derived from the yeast expression system [16,20,30,32,34]. In this study, we demonstrated the pH-dependent iron-binding activity of rpLF, showcasing its highest iron-binding capacity near physiological pH (Figure 3). In contrast to commercial bLF, which maintains identical iron-binding capacity at both pH 7 and pH 11, rpLF exhibited a reduction of about 70% in iron binding at pH 11 compared to pH 7, indicating higher susceptibility to an extremely alkaline environment.

LF and its derived peptides exhibit significant antimicrobial effects against various pathogens, including *Escherichia*, *Pseudomonas*, *Bacillus*, *Staphylococcus*, *Klebsiella*, *Listeria*, *Salmonella*, *Candida*, and *Aspergillus* [16,35,36,37]. In this study, we presented noteworthy antimicrobial effects of rpLF against *E. coli*, *S. aureus*, and *C. albicans* (Figure 5 and Table 2). Our data indicate that rpLF has a comparable minimum inhibitory concentration (MIC) and minimum bactericidal concentration (MBC) to commercial bLF, and its hydrolysate (rpLFH) is more effective than the intact protein. Several LF-derived antimicrobial peptides have been identified to date, such as lactoferricin (LFcin) [38] and lactoferrampin [39]. Earlier, we elucidated the antimicrobial effects of a series of synthetic porcine, bovine, and human LFcins against *E. coli*, *S. aureus*, and *C. albicans*. Our data revealed that porcine LFcin exhibited MIC and MBC values ranging from 32 to 64 μg/mL, surpassing human LFcin but slightly trailing bovine LFcin [40]. Although LFcin can be generated by pepsin digestion [38], confirmation of LFcin presence in rpLFH requires further verification through mass spectrometry.

In vivo, LF has demonstrated anticancer activities in rat models bearing diverse tumors, including lung, esophagus, liver, and colorectal cancers [41,42,43]. In vitro, LF exerts its anticancer effects by modulating the cell cycle, inducing apoptosis, and inhibiting migration, invasion, and metastasis in various human cancer cell lines, such as lung cancer (A549), breast cancer (MDA-MB-231 and MCF-7), oral squamous cell carcinoma (HSC-2, HSC-3, and HSC-4), stomach cancer (SGC-7901), and liver cancer (HepG2) [41,42,43,44,45,46,47,48]. Typically, these studies utilized either bLF or hLF. This study reports, for the first time, the anticancer effect of yeast-produced rpLF, demonstrating the inhibition of cell viability and promotion of apoptosis in A549, MDA-MB-231, and Hep3B cells, with rpLFH exhibiting more potent in vitro anticancer effects than intact rpLF (Figure 6). Surprisingly, although previous studies demonstrated the anticancer effects of LF and LFcin on HepG2, corresponding results have not been reported for Hep3B, possibly due to distinct pharmacological differences between HepG2 and Hep3B [48]. It is worth noting that rpLFH at 1 mg/mL caused a more than 50% reduction in cell viability for the tested cancer cells, suggesting its IC_50_ index (48 h treatment) is around this concentration. The measurement of the IC_50_ index for bLF and rpLF was not conducted in the present study, but it can be anticipated to be several times higher than that of rpLFH. Our previous data can support this speculation by finding that bLF at a concentration of 7.5 mg/mL caused a more than 50% reduction in the viability of A549 and CL1-0 cells as compared to Beas 2B cells [44]. This also hints at a similar IC_50_ index for rpLF against these cancer cells. In the future, the IC_50_, i.e., selectivity index [49], for rpLF or its derived peptides should be precisely determined in vitro to provide more information for in vivo studies or clinical applications.

In conclusion, this study represents the inaugural use of a glucose-inducible *G1* promoter for high-level production of rpLF in *P. pastoris*. The procedures for rpLF expression and purification are both feasible and cost-effective, and the iron-binding, antimicrobial, and anticancer functions of rpLF are confirmed. Unlike the pharmaceutical applications of bLF and hLF, the demand for rpLF in animal health is anticipated, necessitating large-scale production. The current yeast expression system can meet this demand and, importantly, is scalable for the production of recombinant proteins with high added value.

## 4. Materials and Methods

### 4.1. Plasmid Construction, Transformation, and High-Copy-Number Clone Selection

Building upon our prior work [28], we generated a novel recombinant pPICZαC-P_G1_-pLF plasmid in this study. This involved replacing the original *AOX1* promoter region (a BglII-HindIII fragment) of the pPICZαC-rPLF plasmid with a *G1* promoter DNA fragment (a BglII-HindIII fragment). This modification aimed at achieving high-level expression of glucose-inducible rpLF protein in yeast culture media (Figure S1A). The coding region for rpLF expression was rigorously validated for sequence accuracy (Figure S1B).

Transformation of pPICZαC-P_G1_-pLF into *P. pastoris* GS115 was carried out using electroporation, and the subsequent selection of Zeocin-resistant yeast transformants followed established protocols from our previous studies [28,50]. The identification of high-copy-number transformants was achieved through slot-blot DNA hybridization [51]. In brief, 10 μg of genomic DNA was blotted onto a nitrocellulose membrane and then subjected to hybridization with a freshly prepared ^32^P-labeled DNA probe at 42 °C for 16–20 h. Subsequently, the membrane underwent washing steps with 2× SSC (with 0.1% SDS) at room temperature (RT), followed by sequential washes with 0.2× SSC (with 0.1% SDS) at 48 °C, 50 °C, 52 °C, and 55 °C, each lasting 15 min. Hybridization patterns were visualized on X-ray film through autoradiography.

### 4.2. Small- and Large-Scale Expression of rpLF

The small-scale culturing was started by inoculating a single colony of the identified high-copy-number clone in 5 mL YPD broth (1% yeast extract, 2% peptone, 2% glucose, and 100 μg/mL Zeocin) and incubating at 30 °C for 3 days with vigorous shaking (230 rpm). The culture was then inoculated into 50 mL of BMGY medium (1% yeast extract, 2% peptone, 0.1 M potassium phosphate (pH 6.0), 1.34% yeast nitrogen base, 1% glycerol, and 1 × 10^−5^% biotin) to further amplify for 16 h. Afterward, aliquots of the broth (10 mL) were transferred into 100 mL of fresh BMGY medium in baffled 500 mL shaker flasks and induced rpLF expression by adding various concentrations of glucose at an interval of 24 h. 

Large-scale production of rpLF was performed using a benchtop Biostat^®^ A Plus fermenter (Sartorius, Gottingen, Germany). Six flasks of 50 mL yeast cultures (in BMGY media) were prepared in advance as per the abovementioned procedures. The fermentation process was started by adding the prepared culture to the fermenter containing 3 L of basal salts medium (26.7 mL/L H_3_PO_4_, 0.93 g/L CaSO_4_, 18.2 g/L K_2_SO_4_, 14.9 g/L MgSO_4_·7H_2_O, 4.1 g/L KOH, and 40 mL/L glycerol). The fermenter was stirred at a regular speed of 800 rpm, the dissolved oxygen (DO) was set at 30 ± 10%, and the pH value was maintained at 6 by adding 20% NH_4_OH. After 48 h, 200 mL of 50% glycerol supplemented with 2.4 mL of PTM1 solution (6 g/L CuSO_4_, 0.08 g/L NaI, 3 g/L MnSO_4_·H_2_O, 0.2 g/L Na_2_MoO_4_·2H_2_O, 0.02 g/L H_3_BO_3_, 0.5 g/L CoCl_2_, 20 g/L ZnCl_2_, 65 g/L FeSO_4_·7H_2_O, 0.2 g/L biotin, and 5 mL/L H_2_SO_4_) was fed at a speed of 0.4 mL/min. The glucose solution (1%) was then added to induce rpLF expression at the onset of 70 h with a feeding speed of 0.3 mL/min until the end of fermentation. During the fermentation process, aliquots of samples were taken at 24 h intervals for the measurement of optical densities at 600 nm (OD_600_) and the total protein secreted in the culture supernatant. At the end, the culture (4.5 L) was harvested and clarified by centrifugation at 8000 rpm (4 °C, 20 min) several times, and the culture supernatant (2.9 L) was then subjected to rpLF purification. 

### 4.3. rpLF Purification

The 2.9 L culture supernatant underwent initial filtration using a 0.45 micron hollow fiber cartridge (110 cm^2^) (GE Healthcare, Chicago, IL, USA). Subsequently, the filtrate was subjected to sequential purification using Biomax^TM^ 100 kD (0.1 m^2^) and 30 kD (0.1 m^2^) cassettes (Millipore, Burlington, MA, USA). Volume changes, total protein concentrations, and the purity and recovery rate of rpLF were assessed at each purification step.

Following tangential-flow ultrafiltration, aliquots of rpLF crudes underwent further sequential purification using a 5 mL HiTrap Heparin column (GE Healthcare) and a Superdex 75 10/300 GL column (Merck, Darmstadt, Germany), both integrated into an AKTA Purifier 10 FPLC system (GE Healthcare). Fractions containing rpLF protein were pooled and desalted using a HiTrap desalting column (GE Healthcare) before functional characterization. Total protein concentration was determined using the BCA method, and rpLF protein concentration was measured by SDS-PAGE and Western blotting, comparing it with a known amount of bLF (Phermpep Co., Taichung, Taiwan).

### 4.4. Reverse Transcriptase–Polymerase Chain Reaction (RT-PCR)

Total RNA was extracted using Trizol solution (Invitrogen, Carlsbad, CA, USA) as per the manufacturer’s instructions. First, 1 μg of total RNA was subjected to the generation of cDNA using the Improm-II reverse transcription system (Promega, Madison, WI, USA), and the subsequent PCR was performed with primer sets for rpLF (5′-ATG TCG CAT TTG TGA GAG ATT-3′ and 5′-CTC GCT TGG GCA TTT GGT T-3′) and yG3PDH (5′-GTA ACA TCA TTC CAT CTT CC-3′ and 5′-TCT TCA GTG TAA CCC AAA AC-3′). The PCR amplification was performed for 35 cycles (94 °C for 30 s, 55 °C for 1 min, and 72 °C for 1 min). Afterward, the PCR products were visualized with a 2% agarose gel.

### 4.5. rpLF Hydrolysate Preparation

The simulated gastrointestinal digestion of rpLF was conducted according to established protocols [38]. Initially, rpLF protein (5 mg/mL) underwent digestion with pepsin (4000 U/mL) at 37 °C and pH 2 for 4 h. Subsequently, the solution was incubated at 80 °C for 15 min to deactivate pepsin. Centrifugation at 15,000× *g* for 2 min was performed to eliminate denatured protein sediment. The resulting solution was then combined with an equal volume of pancreatin solution (1% pancreatin and 50 mM potassium phosphate, pH 7.5) and allowed to react at 37 °C for 2 h. This was followed by an 80 °C inactivation step for 15 min and another round of centrifugation to remove denatured protein sediment. The above in vitro digestion was performed in triplicate. Throughout the process, samples were periodically withdrawn for HPLC-based digestion monitoring [52], and the prominent peak of the hydrolysate (rpLFH) was collected using an autocollector.

### 4.6. Iron (Fe^3+^)-Binding Assay

The iron-binding assay was conducted following a modified protocol as previously described [30]. In brief, rpLF or bLF was introduced into a Fe^3+^-containing buffer (25 mM Tris, 10 mM NaHCO_3_, and 300 μM FeCl_3_), and the pH was adjusted, allowing binding to occur at 37 °C for 30 min. Subsequently, iron-bound protein and unbound Fe^3+^ were separated using a Bio-Gel P-6 column (Bio-Rad, Hercules, CA, USA). Absorption spectra of the iron-bound protein samples were scanned across wavelengths ranging from 280 nm to 680 nm. The iron-binding ratios were determined by comparing the OD_500_ of Fe^3+^-bound protein samples with those of the initial Fe^3+^-containing buffer.

### 4.7. Microorganisms and Cell Lines

Microbial strains, including *Escherichia coli* ATCC 25922, *Staphylococcus aureus* ATCC 25923, and *Candida albicans* ATCC 14053, were obtained from the Taiwan Bioresource Collection and Research Center (Hsinchu, Taiwan). *E. coli* was cultured on nutrient agar (0.3% beef extract, 0.5% peptone, and 1.5% agar) at 37 °C, *S. aureus* on tryptic soy agar (1.5% tryptone, 0.5% soytone, 0.5% NaCl, and 1.5% agar) at 37 °C, and *C. albicans* on YM agar (0.3% yeast extract, 0.3% malt extract, 0.5% peptone, 1% dextrose, and 2% agar) at RT.

Human cancer cell lines A549 (lung cancer), MDA-MB-231 (triple-negative breast cancer), and Hep3B (liver cancer) were provided by Professor Chia-Che Chang from the Institute of Biomedical Science, National Chung Hsing University, Taichung, Taiwan. These cells were cultured in DMEM or RPMI-1640 medium supplemented with 10% fetal bovine serum (FBS), 2 mM L-glutamine, and 1% penicillin/streptomycin in a humidified incubator (37 °C, 5% CO_2_).

### 4.8. Antimicrobial Assay and Microscopic Observation

Antimicrobial assays were conducted following established protocols [50]. In brief, standard solutions containing 1 × 10^6^ colony-forming units (CFUs)/mL of *E. coli*, *S. aureus*, and *C. albicans* were prepared in peptone water (2% peptone and 1% NaCl, pH 6.8). For MIC determination, an inoculum of 1 × 10^5^ CFUs was combined with different concentrations of bLF, rpLF, and rpLFH (7.5–960 μg/mL), followed by incubation at 37 °C for 16 h. MIC was defined as the lowest concentration inhibiting microbial growth. For MBC determination, aliquots of the solutions were spread onto agar plates and incubated at 37 °C overnight. MBC was defined as the concentration reducing microbial growth by 99.9%. These experiments were conducted in triplicate.

Additionally, an inoculum of 2 × 10^5^ CFUs was mixed with an equal volume of 5 mg/mL bLF or rpLF or 500 μg/mL rpLFH at 37 °C for 0–3 h. Subsequently, samples were fixed, coated, and visualized using a scanning electron microscope (JEOL, Tokyo, Japan).

### 4.9. Cell Viability and Apoptotic Assays

Cell viability assays were conducted using the WST-1 reagent (Abcam, Cambridge, MA, USA). Cells (2 × 10^4^/well) were seeded onto 96-well plates and treated with bLF (5 mg/mL), rpLF (5 mg/mL), or rpLFH (500 μg/mL) for 48 h. Subsequently, WST-1 (10 μL/well) was added and incubated for 2 h, and the absorbance was measured at 440 nm against a background control. Additionally, 2 × 10^5^ cells were seeded onto 12-well plates and subjected to the same treatments. Cells were then collected for apoptotic assays using an Annexin V-APC/7-AAD apoptosis detection kit (Elabscience, Houston, TX, USA) following the manufacturer’s instructions. Samples from the apoptotic assays were analyzed using a flow cytometer (BD Biosciences, San Jose, CA, USA).

### 4.10. Statistical Analysis

Data are presented as mean ± SD and were subjected to analysis using two-way ANOVA followed by Dunnett’s multiple comparison test. Statistical significance was considered for *p*-values less than 0.05 in comparison to the PBS treatment.

## Figures and Tables

**Table 1:** Stepwise purification of rpLF from a 4.5 L fed-batch yeast culture by tangential-flow ultrafiltration.

Step	Start Volume (mL)	End Volume (mL)	Total Protein (mg/mL)	rpLF (mg/mL)	Purity (%)	Recovery (%)
Hollow fiber cartridge 0.45 micron	2900	2400	8.3	4.3	52.4	100
Biomax 100 kD	2400	1350	10.8	7.3	67.9	95.5
Biomax 30 kD	1350	240	27.3	23.1	84.9	53.7

**Table 2:** The minimal inhibitory and bactericidal concentrations of bLF, rpLF, and rpLFH against *E. coli*, *S. aureus*, and *C. albicans*.

Protein	*E. coli*		*S. aureus*		*C. albicans*	
	MIC	MBC	MIC	MBC	MIC	MBC
Empty	>960	>960	>960	>960	>960	>960
bLF	480	>960	720	>960	480	>960
rpLF	720	>960	960	>960	720	>960
rpLFH	120	240	180	360	240	480

MIC: minimal inhibitory concentration; MBC: minimal bactericidal concentration; unit: μg/mL.

## Data Availability

The data presented in this study are available on request from the corresponding author.

## Supplementary Materials

The following supporting information can be downloaded at: https://www.mdpi.com/article/10.3390/ijms25031818/s1.
