# Supplementary material for: Production of Bioactive Porcine Lactoferrin through a Novel Glucose-Inducible Expression System in Pichia pastoris: Unveiling Antimicrobial and Anticancer Functionalities
Source: Int J Mol Sci. 2024 Feb 2;25(3):1818. doi: 10.3390/ijms25031818 (PMC10855427; doi:10.3390/ijms25031818)
Supplement: Supplementary file 1 [file ijms-25-01818-s001.zip › ijms-2807797-supplementary.pdf]

Supplementary

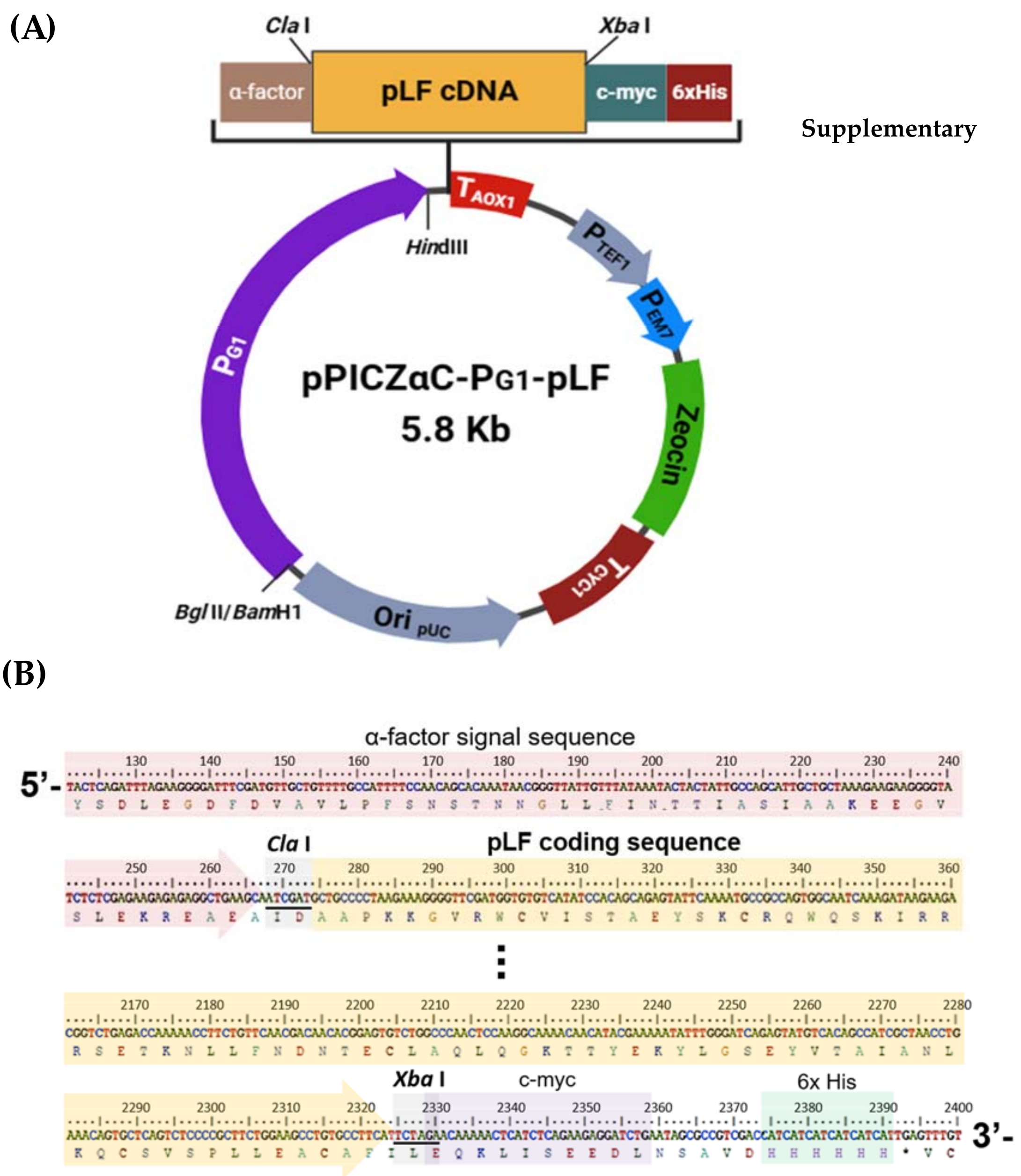

Figure S1. Recombinant glucose-inducible plasmid for rpLF expression in *Pichia pastoris*. (**A**) Schematic representation of the recombinant pPICZ*α*C-P$_{G1}$-pLF plasmid. In this study, the *AOX1* promoter region (a *Bgl*II-*Hind*III fragment) from the previous pPICZ*α*C-rPLF plasmid [28] was substituted with the *G1* promoter (P$_{G1}$) (a *Bam*HI-*Hind*III fragment). (**B**) 5′- and 3′-end flanking sequences of the pLF cDNA insert. The sequences for *α*-factor, pLF, and the *Cla*I and *Xba*I insertional sites, along with sequences for c-myc and 6×His epitopes, are depicted as indicated.

(A)

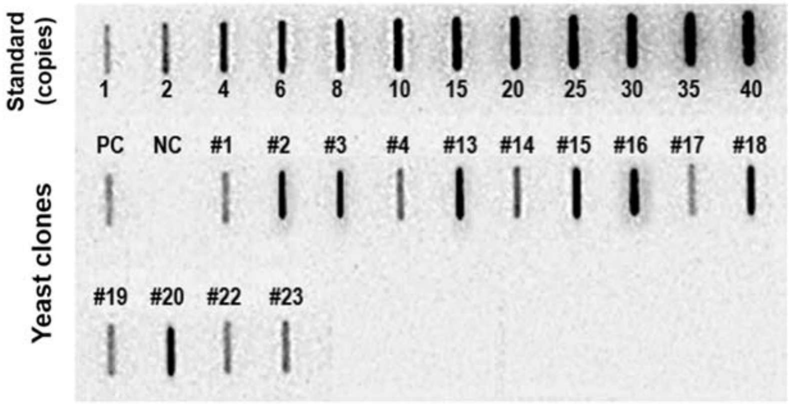

(B)

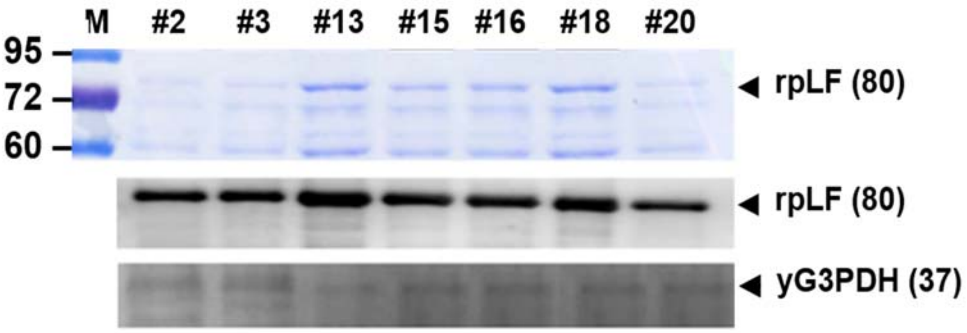

Supplementary Figure S2: Screening of pPICZαC-P_{G1}-pLF transformed yeast clones. (**A**) Identification of high-copy-number transformed yeast clones using slot blot analysis. (**B**) Characterization of rpLF expression in the identified high-copy-number clones through SDS-PAGE and Western blotting. Clone 13 was selected for large-scale production of rpLF in this study.
